# Ureteral stent encrustation in an HIV patient treated with disposable flexible ureteroscope: a case report

**DOI:** 10.11604/pamj.2024.47.145.39961

**Published:** 2024-03-27

**Authors:** Lin Xiong, Nga-nuen Loo, Zhen-Quen Lu, Xiang Xu, Genggeng Wei

**Affiliations:** 1 Department of Urology, The University of Hong Kong-Shenzhen Hospital, Shenzhen, China,; 2 International School, Jinan University, Guangzhou, China

**Keywords:** Human immunodeficiency virus, urolithiasis, ureteroscope, case report

## Abstract

Human immunodeficiency virus prevalence was increasing worldwide. Medication-associated urinary calculi are very commonly caused by medications used to treat HIV-positive patients. We present a case of an HIV-positive 39-year-old male with ureteral stent encrustation and kidney stone. Ureterolithotripsy using a disposable flexible ureteroscope is performed. The postoperative evolution was favorable. The disposable flexible ureteroscope is effective in the treatment of HIV combined with ureteral stent encrustation.

## Introduction

The global prevalence of human immunodeficiency virus (HIV) was increasing worldwide. Urolithiasis is a common urological disease and the procedure of ureteral stent implantation is often performed after its management. The number of HIV-positive patients with calcified ureteral stents is increasing every year. When ureteral stents are indwelling for an extended length of time, some of them become calcified, causing issues such as extraction difficulties [1]. We report a case of urethral stent encrustation combined with a kidney stone in an HIV patient.

## Patient and observation

**Patient information:** we present a case of a 39-year-old male patient, HIV-positive, non-smoker, and non-alcoholic.

**Clinical findings:** the patient is asymptomatic. He had no pain, no fever, no nausea, and no vomiting. He had neither a renal infection nor blood in the urine. The patient´s renal function is not affected. During physical examination, the patient only showed costovertebral angle tenderness. The rest of the examination findings are unremarkable.

**Timeline of the current episode:** in 2020, the patient was diagnosed with HIV-positive and started treatment for HIV. In March 2021, the left laparoscopic ureterolithotomy and urethral stenting were performed. In October 2021, there was a follow-up after surgery. Urological computed tomography (CT) was performed. Phase one of left transurethral lithotripsy and urethral stent replacement were performed. In November 2021, phase two of left transurethral lithotripsy and urethral stent replacement were performed. Renal stone analysis was performed. In December 2021, the urological CT was performed and the urethral stent was removed.

**Figure 1:**
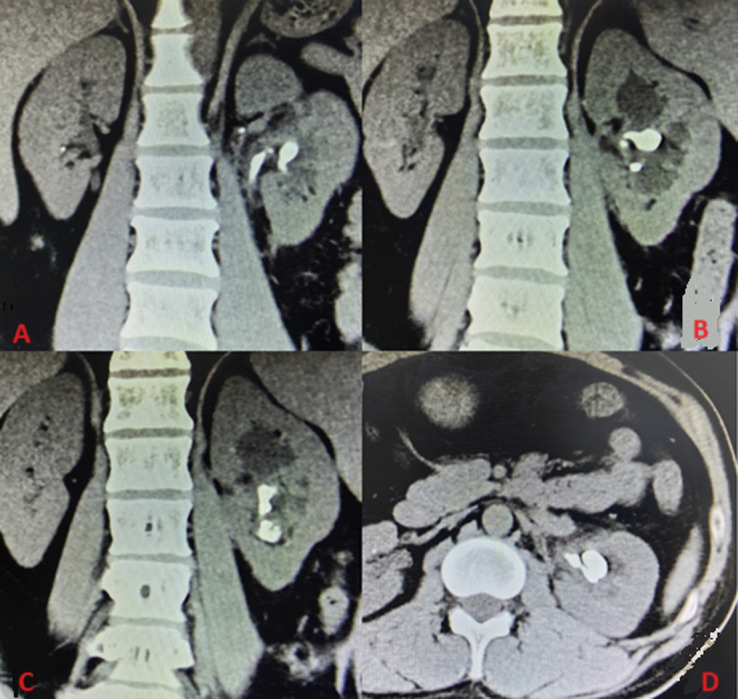
computed tomography showing ureteral stent encrustation and kidney stones; A,B) ureteral stent and ureteral stent encrustation; C) kidney stones located in the renal pelvis and subrenal calyx; D) kidney stones of CT cross-sectional imaging

**Diagnostic assessment:** the patient´s CT+T lymphocyte count was ≥ 200 cells/μl and the HIV load is > 500. Urological computed tomography was performed. It showed that there is a retained ureteral stent calcification and a left kidney stone. The size of the calcification on the left ureteral stent is 16 mmx11 mm, with a CT maximum value of 1485 and a mean value of 1155 (Figure 1).

**Diagnosis:** a diagnosis of ureteral stent encrustation and kidney stone was made.

**Figure 2:**
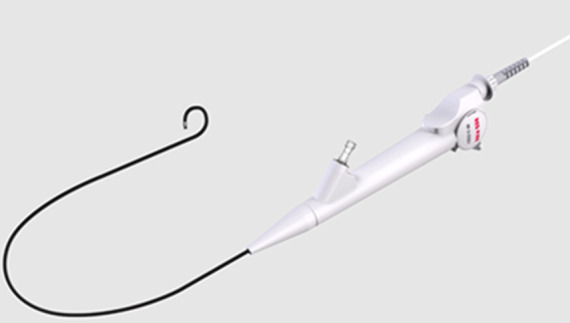
disposable red pine flexible ureteroscope; the single-used flexible urethroscope used during the surgery

**Figure 3:**
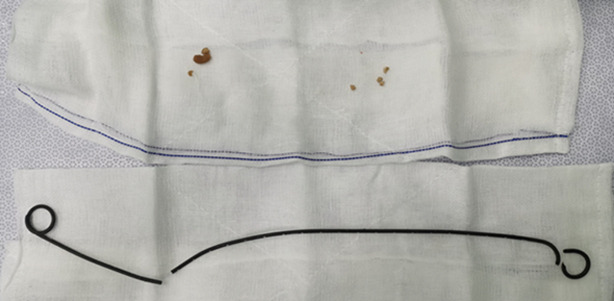
the segment of the ureteral stent obtained after the surgery; it is broken into three parts

**Therapeutic interventions:** after thorough examinations, the patient underwent transurethral left ureteral flexible lithotripsy and left ureteral stent replacement. Intraoperatively, there was no obvious calcification in the bladder segment and the ureteral segment of the ureteral stent. These segments of the ureteral stent were cut with a holmium laser with the power set to 1.2j/5hz and then removed in sections. Then, the ureteral access sheath was inserted and adjusted to slightly below the pelvic. Redpine disposable flexible ureteroscope (Figure 2) was then positioned. the remaining ureteral stent was found to have obvious calcification. The stones were fragmented with the holmium laser power was set to 0.6j/50hz. The remaining ureteral stents were removed using a lithotripsy extraction basket (Figure 3). Due to the significant stone load, a second operation was performed. The surgery was carried out one month later with transurethral left ureteral flexible lithotripsy and replacement of the left ureteral stent. This operation also utilizes the red pine disposable flexible ureteroscope. During this operation, the residual kidney stones were fragmented and the residual stones bigger than 2 mm were removed using a lithotripsy extraction basket. The stones from both surgeries were analyzed as a mixture of calcium oxalate dihydrate and calcium hydrogen phosphate dihydrate. A 5F polaris ultra ureteral stent was placed after both procedures.

**Follow-up and outcome of interventions:** a urological CT was performed and suggested no residue stone. The urethral stent was then removed.

**Patient perspective:** “I was quite worried that my HIV status would contaminate the tools and harm other patients or medical personnel. However, I was rather relieved to learn that they would be utilizing urethroscopes that could only be used once.”

**Informed consent:** the patient gave a written informed consent.

## Discussion

Ureteral stents are generally routinely used in urology, whether for flexible ureteroscopic lithotripsy, percutaneous nephrolithotripsy, or laparoscopic ureterotomy for stone extraction. In clinical practice, calcification is typical following protracted stent installation. Various variables, such as stent material and bacterial colonization, can influence stent calcification, but the most significant risk factor is the period of ureteral stent retention. Kawahara [2] in their study, reported stent encrustation rates of 26.8% at less than 6 weeks, 56.9% at 6 to 12 weeks, between 6 to 12 weeks, and 75.9% at more than 12 weeks of indwelling time. In our case, the patient had a ureteral stent indwelling for more than 6 months and showed calcification on the pelvic segment of the ureteral stent. There are several different clinical criteria for grading ureteral stent calcification. According to the FECal classification [3], our case belongs to grade 3. According to the KUB criteria [4], our case has a K score of 4, U score of 1, and a B score of 1, with a total of 6. According to Singh I classification [5], our case is classified as mild encrusted. Our case was categorized as mild encrusted based on all three classification criteria. Cicione A [6] and other researchers recommended the KUB criteria as a better predictor of the requirement for numerous procedures.

A tiered approach to surgery is advised because ureteral stent tube calcification typically requires severe treatments. An average of 3±1.08 operations per patient was reported by Thangavelu M *et al*. [7] for 13 cases. In our case, the first procedure was primarily to remove the calcification ureteral stent, and the second operation was to treat any remaining kidney stones.

There is no conventional therapy for ureteral stent calcification, which may generally be addressed by several combinations of surgical techniques, depending on the location of the calcification and the degree of calcification. Thangavelu M *et al*. [7] reported 13 management modalities for ureteral stent calcification: eight that involved bladder lithotripsy, ureteroscopy (URS), and retrograde intrarenal surgery (RIRS), two that involved URS and extracorporeal shock wave lithotripsy (ESWL), two that involved bladder stone, URS, RIRS, and ESWL. According to Torricelli FC's report [8], there was a considerably higher chance of bleeding in HIV patients who underwent percutaneous nephrolithotomy for upper urinary tract stones. This encompasses not just the patient's danger, but also the risk to the health care practitioner. Ureteroscopic flexible surgery minimizes the risk of occupational exposure to the surgeon by eliminating intraoperative injuries as there are no edges or sharp tools etc. In our case, we used the combination of URS and RIRS for the therapy of ureteral stent calcification, attaining clinical effectiveness based on effective risk reduction to the health care worker.

Transurethral flexible lithotripsy was previously performed using a conventional reusable flexible ureteroscope (ru-fURS). One in eight of the ru-fURS were discovered to be microbiologically contaminated, according to Legemate JD *et al*. [9]. The insufficient sterilization process of ru-fURS was documented by Ofstead CL *et al*. [10], who also discovered contamination in 100% of the sterilized ru-fURS, as well as microbial growth in 13% of them and hemoglobin in 63%. The ru-fURS had hemoglobin contamination levels above the threshold for a thoroughly cleaned gastrointestinal endoscopy. The use of a disposable flexible ureteroscope for ureterolithotripsy in HIV patients eliminates blood contact, one of the mechanisms by which HIV is spread, and also speeds up the sterilization procedure. This is also one of the key reasons why we use disposable ureteroscopes to treat such HIV diseases.

## Conclusion

The disposable flexible ureteroscope is effective in the treatment of HIV-combined ureteral stent encrustation. Not only can it reduce the likelihood of HIV cross-contamination. When compared to ru-fURS, it may also be less cost-effective.
